# Vitamin D deficiency and pain severity in endometriosis: a phenotype-based retrospective observational study

**DOI:** 10.1186/s12905-026-04542-8

**Published:** 2026-05-28

**Authors:** Ismail Baglar, Fatih Sanlikan, Esra Keles, Aslı Sasmaz, Kubra Karakas Soylu, Murat Api

**Affiliations:** 1 Department of Obstetrics and Gynecology, University of Health Sciences Turkey, Kartal Lütfi Kırdar City Hospital, Istanbul, 34668 Turkey; 2 Department of Gynecologic Oncology, University of Health Sciences Turkey, Kartal Lütfi Kırdar City Hospital, Istanbul, 34668 Turkey

**Keywords:** Endometriosis, Vitamin D, Deep infiltrating endometriosis, Ovarian endometrioma, Biomarker

## Abstract

**Objective:**

To evaluate the association between serum 25-hydroxyvitamin D [25(OH)D] levels and pain severity in women with surgically and histopathologically confirmed endometriosis, and to assess whether this association differs between ovarian endometrioma (OE) and deep infiltrating endometriosis (DIE) phenotypes.

**Methods:**

This retrospective observational analytical study included 427 women with surgically confirmed endometriosis. Patients were classified according to dominant phenotype as OE (n=231) or DIE (n=196). Serum 25(OH)D levels were categorized as deficient (<20 ng/mL), insufficient (20–30 ng/mL), and sufficient (≥30 ng/mL). Pain severity—including dysmenorrhea, chronic pelvic pain, and dyspareunia—was assessed using the Visual Analog Scale (VAS). Multivariable linear regression analyses were performed to examine the association between vitamin D levels and pain scores, adjusting for age, body mass index, and seasonal variation.

**Results:**

Serum 25(OH)D levels were inversely associated with all pain parameters (p<0.001). Patients with vitamin D deficiency had higher median VAS scores compared to those with sufficient levels [7.0 (6.0–8.0) vs 4.0 (3.0–5.0), p<0.001]. In multivariable analyses, lower 25(OH)D levels remained significantly associated with higher pain severity (β = -0.28, p<0.001). A significant interaction between vitamin D levels and disease phenotype was observed (interaction p=0.004), suggesting that the association between lower vitamin D levels and higher pain scores was more pronounced in patients with the DIE phenotype compared to those with OE. A threshold value of 18.5 ng/mL demonstrated 74% sensitivity for identifying patients with severe pain (VAS ≥7).

**Conclusion:**

Lower serum vitamin D levels are significantly associated with greater pain severity in women with endometriosis, with a stronger association observed in the DIE phenotype. However, given the retrospective and cross-sectional nature of the analysis, causality cannot be inferred. Prospective studies are needed to further clarify these associations and their potential clinical implications.

## Introduction

Endometriosis is a chronic inflammatory condition affecting approximately 10% of women of reproductive age and is commonly associated with pelvic pain. Dysmenorrhea, chronic pelvic pain, and dyspareunia significantly impair patients’ quality of life. In addition, diagnostic delays—often ranging from 5 to 10 years—further increase the clinical burden of the disease [1].

Although the pathogenesis of endometriosis remains incompletely understood, accumulating evidence suggests that inflammation, immune dysregulation, and oxidative stress play important roles. While the theory of retrograde menstruation provides a partial explanation, the fact that not all exposed individuals develop the disease highlights the contribution of individual biological susceptibility [2, 3].

Vitamin D has recently gained attention in this context. Beyond its classical role in calcium metabolism, vitamin D has been shown to exert anti-inflammatory, immunomodulatory, and antiproliferative effects, suggesting a possible role in endometriosis-related biological processes. The presence of vitamin D receptors in endometriotic tissues further supports this hypothesis [4]. However, findings regarding the relationship between serum 25-hydroxyvitamin D [25(OH)D] levels and endometriosis are inconsistent. While some studies have reported associations between lower 25(OH)D levels and disease presence or severity, others have not demonstrated a significant relationship [5, 6].

With respect to pain, vitamin D has been proposed to be associated with symptom severity, potentially through modulation of inflammatory pathways. However, clinical findings remain conflicting, particularly regarding dysmenorrhea and dyspareunia [7]. Importantly, endometriosis is a heterogeneous disease, and clinical presentation—including pain characteristics—varies across different phenotypes, such as ovarian endometrioma (OE), deep infiltrating endometriosis (DIE), and superficial peritoneal disease. Despite this heterogeneity, most previous studies have not adequately accounted for phenotypic variation, limiting the interpretability of their findings [8].

Among these phenotypes, deep infiltrating endometriosis (DIE) is often considered the most severe form, characterized by deep tissue invasion, increased inflammatory activity, and a strong association with severe pain symptoms. The proximity of DIE lesions to pelvic nerves, along with increased neuroangiogenic activity, may contribute to its distinct pain profile.

Therefore, the aim of this study was to evaluate the association between serum 25(OH)D levels and pain severity in women with endometriosis and to explore whether this association differs according to disease phenotype. By incorporating a phenotype-based approach, this study aims to provide a more nuanced interpretation of previously inconsistent findings and to contribute to the existing literature.

## Materials and methods

### Study design and patient selection

This study was conducted as a single-center retrospective observational analytical study with a cross-sectional assessment of exposure and outcomes. Patients who underwent laparoscopic surgery for suspected endometriosis and had a histopathologically confirmed diagnosis between January 2014 and December 2024 were included. Patients who underwent laparotomy or diagnostic procedures without definitive surgical treatment were excluded to reduce potential heterogeneity in pain perception. All data were retrospectively obtained from the hospital information management system and electronic medical records.

Inclusion criteria were age between 18 and 45 years, surgically and histopathologically confirmed endometriosis, availability of preoperative serum 25-hydroxyvitamin D [25(OH)D] measurements, and documented preoperative pain assessment using the Visual Analog Scale (VAS).

Exclusion criteria included systemic diseases affecting vitamin D metabolism (e.g., chronic kidney disease, liver disease, malabsorption syndromes), active inflammatory or infectious conditions, and use of medications or supplements influencing vitamin D or bone metabolism. Specifically, patients who had received high-dose vitamin D supplementation within the past 6 months, those using multivitamins exceeding 400 IU/day, calcium supplements, glucocorticoids, antiepileptic drugs, or hormonal therapies (including oral contraceptives and GnRH analogs) were excluded. Patients with incomplete or inconsistent data were also excluded.

Demographic and clinical data—including age, body mass index (BMI), obstetric history, surgical findings, and disease stage—were recorded using a standardized data collection form. Disease staging was performed according to the revised American Society for Reproductive Medicine (rASRM) classification.

### Variable definitions and measurements

Serum 25(OH)D levels were obtained from venous blood samples collected preoperatively (08:00–10:00, after at least 8 h of fasting) and measured using a standardized immunoassay method in a central laboratory under uniform conditions.

To account for seasonal variation, sampling dates were recorded and categorized as Winter/Spring versus Summer/Fall. Seasonal variation was initially included as a potential confounder in multivariable analyses and was excluded from the final model as it did not significantly affect the results.

Serum 25(OH)D levels were classified according to Endocrine Society guidelines as deficiency (< 20 ng/mL), insufficiency (20–30 ng/mL), and sufficiency (≥ 30 ng/mL). Vitamin D levels were analyzed both as continuous and categorical variables.

Pain severity was assessed preoperatively using the Visual Analog Scale (VAS, 0–10 cm) separately for dysmenorrhea, chronic pelvic pain, and dyspareunia. Each pain domain was analyzed as an independent outcome, and no composite or total VAS score was used in the primary analyses [9].

Analgesic use was recorded as an additional descriptive variable reflecting clinical burden and categorized according to frequency (none, intermittent, regular) and type (nonsteroidal anti-inflammatory drugs and opioids).

Potential confounders were predefined based on clinical relevance and prior literature and included age, BMI, rASRM stage, and serum CA125 levels.

### Phenotype definition

Endometriosis phenotypes were classified based on surgical and histopathological findings according to established criteria. Deep infiltrating endometriosis (DIE) was defined as lesions infiltrating ≥ 5 mm beneath the peritoneal surface and involving surrounding structures. Ovarian endometrioma (OE) was defined as isolated ovarian cystic lesions without evidence of DIE.

In cases where multiple phenotypes coexisted, a hierarchical classification approach was applied, with priority given to DIE as the dominant phenotype due to its greater clinical severity. Therefore, patients with both OE and DIE were categorized in the DIE group. Mixed phenotypes were not analyzed as a separate category.

### Statistical analysis

Data distribution was assessed using the Kolmogorov–Smirnov test and Q–Q plots. Continuous variables were presented as mean ± standard deviation or median (interquartile range), and categorical variables as frequencies and percentages. Group comparisons were performed using the independent samples t-test or Mann–Whitney U test, as appropriate.

Chronic pelvic pain (VAS score) was defined a priori as the primary outcome. Dysmenorrhea and dyspareunia were analyzed as secondary outcomes. Separate multivariable linear regression models were constructed for each pain outcome. Variables included in the multivariable models were selected based on both statistical and clinical considerations. Specifically, variables with *p* < 0.10 in univariate analyses, along with clinically relevant covariates (age, BMI, rASRM stage, and CA125), were entered into the models.

Assumptions of linear regression—including linearity, normality of residuals, homoscedasticity, and absence of multicollinearity—were formally evaluated. Multicollinearity was assessed using the Variance Inflation Factor (VIF), with values < 3 considered acceptable. To assess whether the association between 25(OH)D levels and pain outcomes differed by phenotype, an interaction term (25(OH)D × phenotype) was included in the models. Receiver operating characteristic (ROC) curve analysis was performed to evaluate the discriminatory ability of 25(OH)D levels for identifying severe pain (VAS ≥ 7). The optimal cutoff value was determined using the Youden Index. All analyses were conducted using SPSS version 26.0 (IBM Corp., Armonk, NY, USA), and a two-sided p-value < 0.05 was considered statistically significant.

## Results

### Patient flow and baseline characteristics

**Fig. 1:**
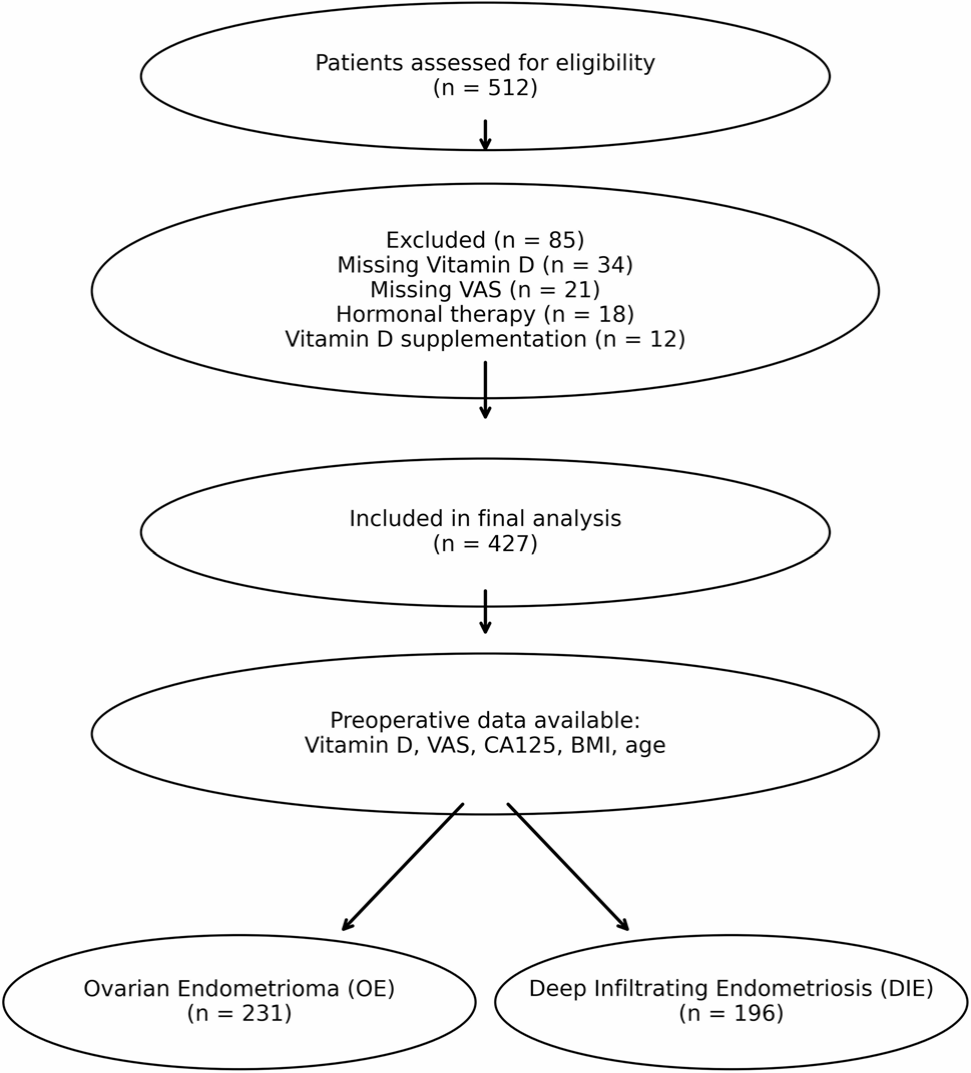
Patient flow diagram of the study population. Flowchart illustrating the selection process of the study population. A total of 512 patients were initially assessed for eligibility. After applying exclusion criteria (missing vitamin D data, missing VAS scores, hormonal therapy use, and vitamin D supplementation), 427 patients were included in the final analysis. The study cohort was subsequently divided into two phenotype groups: ovarian endometrioma (OE, *n* = 231) and deep infiltrating endometriosis (DIE, *n* = 196)

A total of 512 patients were screened during the study period, of whom 85 were excluded based on predefined criteria [missing data (vitamin D level, *n* = 34; missing VAS score, *n* = 21), use of hormonal therapy (*n* = 18), and vitamin D supplementation (*n* = 12)]. The final analysis included 427 patients, comprising 231 patients in the ovarian endometrioma (OE) group and 196 in the deep infiltrating endometriosis (DIE) group. The patient selection process is illustrated in Fig. 1.

Baseline demographic and clinical characteristics are presented in Table 1. The OE and DIE groups were comparable in terms of age, body mass index (BMI), and obstetric characteristics (*p* > 0.05). Advanced-stage disease (rASRM Stage III–IV) was more frequent in the DIE group (78.5%) compared to the OE group (*p* = 0.01).

**Table 1:** Baseline Demographic and Clinical Characteristics of Patients According to Endometriosis Phenotype

Variable	OE (*n* = 231)	DIE (*n* = 196)	*p*-value
Age (years)	31.8 ± 5.4	32.6 ± 5.7	0.12
BMI (kg/m²)	25.7 ± 3.8	26.1 ± 4.1	0.28
Gravidity	1 (0–3)	1 (0–2)	0.34
Parity	1 (0–2)	1 (0–2)	0.41
rASRM stage (III–IV, %)	144 (62.3)	154 (78.5)	0.01
Vitamin D (ng/mL)	19.6 ± 7.8	17.2 ± 6.9	0.02
CA125 (U/mL)	54 (28–96)	71 (35–128)	0.01

Continuous variables are presented as mean ± standard deviation or median (interquartile range, IQR), as appropriate. Categorical variables are expressed as number (percentage). Comparisons between groups were performed using appropriate parametric or non-parametric tests based on data distribution

*Abbreviations BMI *body mass index, *OE* ovarian endometrioma, *DIE* deep infiltrating endometriosis, *rASRM* revised American Society for Reproductive Medicine, *CA125* cancer antigen 125

Mean serum 25-hydroxyvitamin D levels were below the deficiency threshold in both groups and were lower in the DIE group (17.2 ± 6.9 vs. 19.6 ± 7.8 ng/mL, *p* = 0.02). CA125 levels were also higher in the DIE group [71 (35–128) vs. 54 (28–96) U/mL, *p* = 0.01].

### Association between vitamin d levels and pain severity

**Fig. 2:**
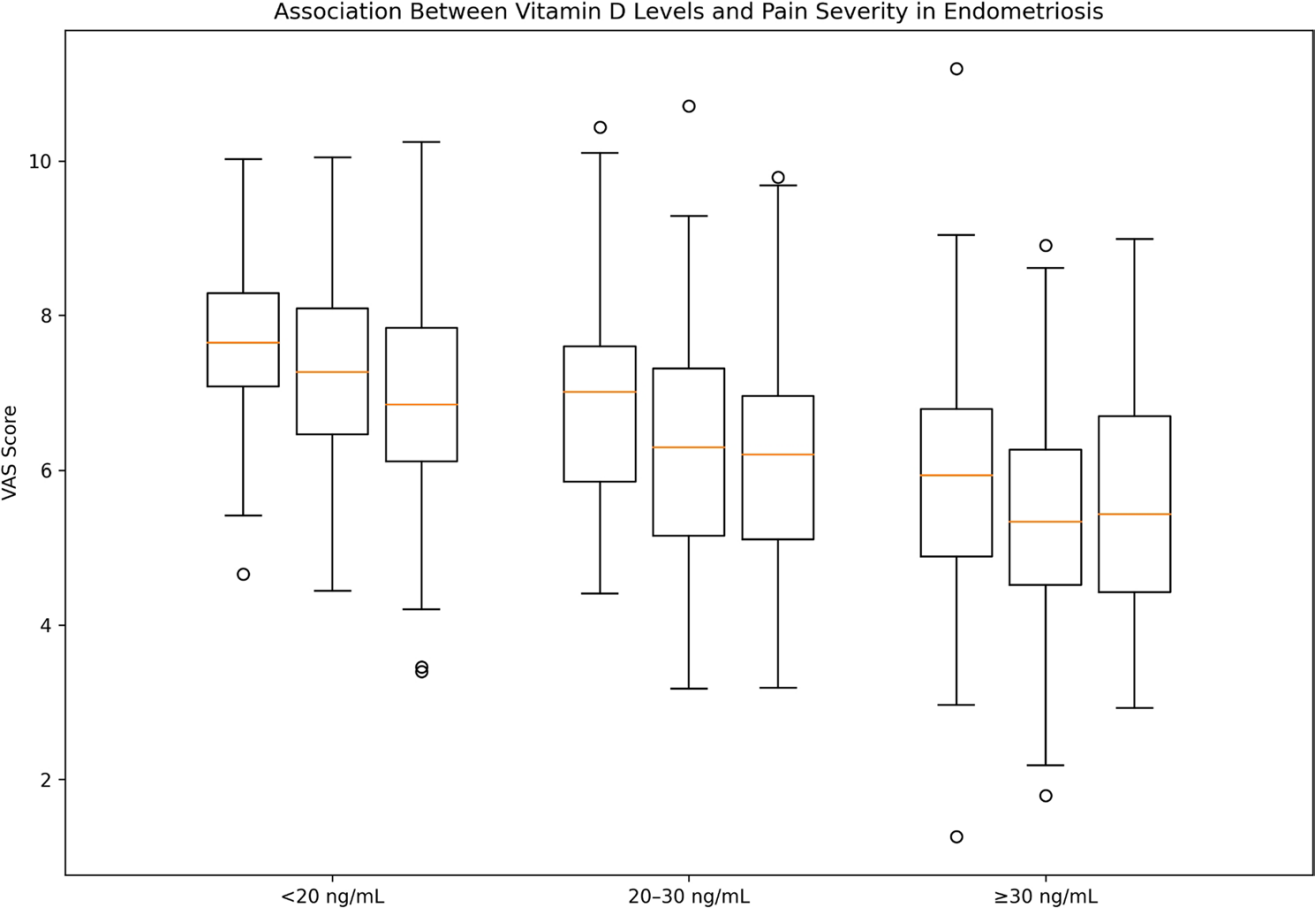
Association between vitamin D levels and pain severity. Boxplot representation of Visual Analog Scale (VAS) scores according to serum 25-hydroxyvitamin D [25(OH)D] categories (< 20 ng/mL, 20–30 ng/mL, ≥ 30 ng/mL). A progressive decrease in pain scores is observed with increasing vitamin D levels across all pain parameters. Median values, interquartile ranges, and outliers are displayed. Lower vitamin D levels were associated with higher pain scores (*p* < 0.001)

Pain scores differed according to vitamin D status across all domains (Table 2; Fig. 2). Patients with vitamin D deficiency (< 20 ng/mL) had higher median VAS scores compared to those with sufficient levels (≥ 30 ng/mL) [7.0 (6.0–8.0) vs. 4.0 (3.0–5.0), *p* < 0.001]. A graded pattern was observed, with lower pain scores at higher vitamin D levels across dysmenorrhea, chronic pelvic pain, and dyspareunia.

**Table 2:** Comparison of Pain Scores and Analgesic Use According to Serum Vitamin D Categories

Variable	< 20 ng/mL (*n* = 198)	20–30 ng/mL (*n* = 143)	≥ 30 ng/mL (*n* = 86)	*p*-value
VAS Dysmenorrhea	7.8 ± 1.6	6.9 ± 1.8	5.8 ± 1.9	< 0.001
VAS CPP	7.2 ± 1.7	6.4 ± 1.9	5.5 ± 2.0	< 0.001
VAS Dyspareunia	6.9 ± 1.8	6.1 ± 1.9	5.2 ± 2.1	< 0.001
Analgesic use, n (%)	163 (82.3)	98 (68.5)	44 (51.2)	< 0.001

Continuous variables are presented as mean ± standard deviation. Comparisons between vitamin D categories were performed using one-way ANOVA for normally distributed variables or the Kruskal–Wallis test for non-normally distributed variables. Post-hoc pairwise comparisons showed that all groups differed significantly from each other (*p* < 0.05)

*Abbreviations VAS*Visual Analog Scale, *CPP* chronic pelvic pain

Analgesic use also varied according to vitamin D status. The prevalence of analgesic use was higher in the vitamin D-deficient group (82.3%) compared to the sufficient group (51.2%) (*p* < 0.001). Regular analgesic use was more frequent among patients with deficiency (68% vs. 28%, *p* < 0.001). A moderate inverse correlation was observed between serum 25(OH)D levels and pain severity (*r* = -0.42, *p* < 0.001).

### Phenotype-based differences in pain

**Fig. 3:**
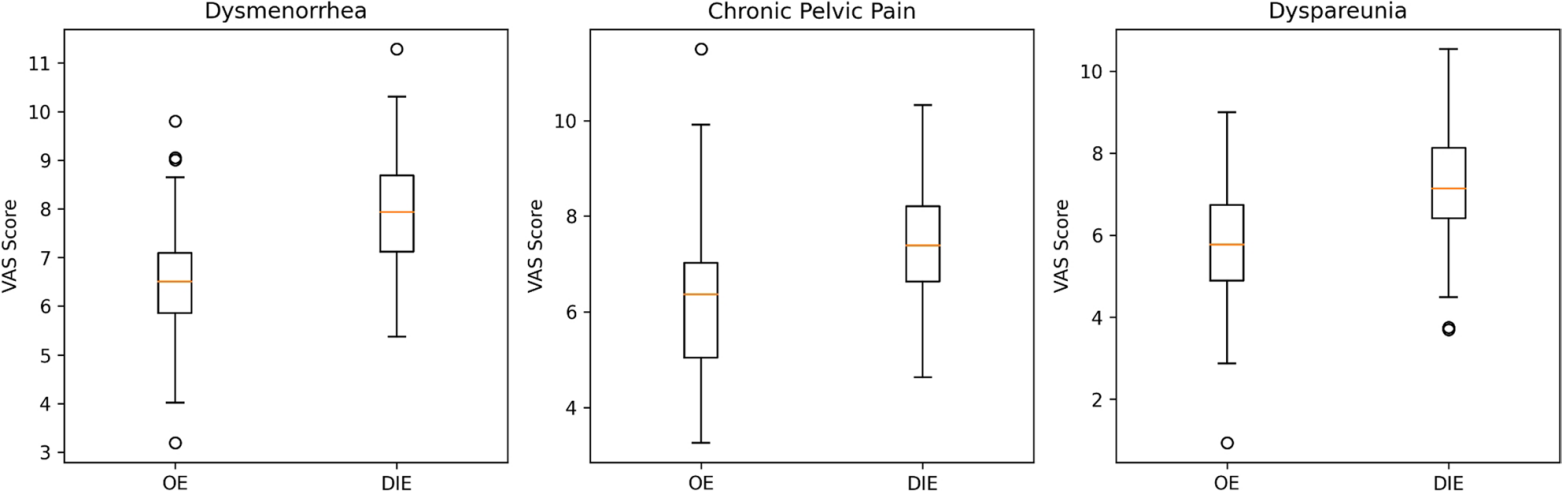
Comparison of pain scores according to endometriosis phenotype. Boxplots comparing VAS scores for dysmenorrhea, chronic pelvic pain, and dyspareunia between ovarian endometrioma (OE) and deep infiltrating endometriosis (DIE) groups. Patients with the DIE phenotype had significantly higher pain scores across all parameters compared to the OE group (*p* < 0.001)

Patients with the DIE phenotype had higher pain scores across all domains compared to those with OE (*p* < 0.001) (Table 3; Fig. 3). The difference was most pronounced for dyspareunia [6.5 (5.0–8.0) vs. 4.0 (3.0–5.5)]. Analgesic use was also more frequent in the DIE group (79.1%) compared to the OE group (*p* < 0.001), consistent with the observed differences in pain scores.

**Table 3:** Comparison of Pain Scores and Analgesic Use Between Endometriosis Phenotypes

Variable	OE (*n* = 231)	DIE (*n* = 196)	*p*-value
VAS Dysmenorrhea	6.6 ± 1.9	7.9 ± 1.5	< 0.001
VAS CPP	6.1 ± 2.0	7.6 ± 1.6	< 0.001
VAS Dyspareunia	5.8 ± 2.1	7.2 ± 1.7	< 0.001
Analgesic use, n (%)	146 (63.2)	155 (79.1)	< 0.001

Continuous variables are presented as mean ± standard deviation. Comparisons between groups were performed using independent samples t-test or Mann–Whitney U test, as appropriate. Categorical variables are expressed as number (percentage) and compared using the chi-square test

*Abbreviations VAS*Visual Analog Scale, * CPP*chronic pelvic pain, * OE *ovarian endometrioma, * DIE*deep infiltrating endometriosis

### Multivariable analysis and interaction effect

In multivariable linear regression analysis adjusting for age, BMI, rASRM stage, and CA125, serum 25(OH)D levels remained significantly associated with chronic pelvic pain (β = -0.28, *p* < 0.001) (Table 4).

**Table 4:** Multivariable Linear Regression Analysis of Factors Associated with Chronic Pelvic Pain

Variable	B (Unstandardized coefficient)	95% CI	*p*-value
Vitamin D (ng/mL)	-0.28	-0.41 to -0.15	< 0.001
Phenotype (DIE vs. OE)	0.34	0.21 to 0.47	< 0.001
Age	0.06	-0.03 to 0.15	0.18
BMI	0.09	-0.02 to 0.20	0.11
rASRM stage	0.22	0.10 to 0.34	0.001
CA125	0.19	0.07 to 0.31	0.003
Vitamin D × Phenotype	-0.17	-0.29 to -0.05	0.004

Multivariable linear regression analysis was performed to evaluate factors associated with chronic pelvic pain severity. Variables were included in the model based on clinical relevance and existing literature. Results are presented as unstandardized regression coefficients (B) with 95% confidence intervals. The interaction term (vitamin D × phenotype) was included to assess effect modification

*Abbreviations VAS*Visual Analog Scale, * BMI*body mass index, *rASRM*revised American Society for Reproductive Medicine,* CI *confidence interval,* OE*ovarian endometrioma, * DIE*deep infiltrating endometriosis

**Fig. 4:**
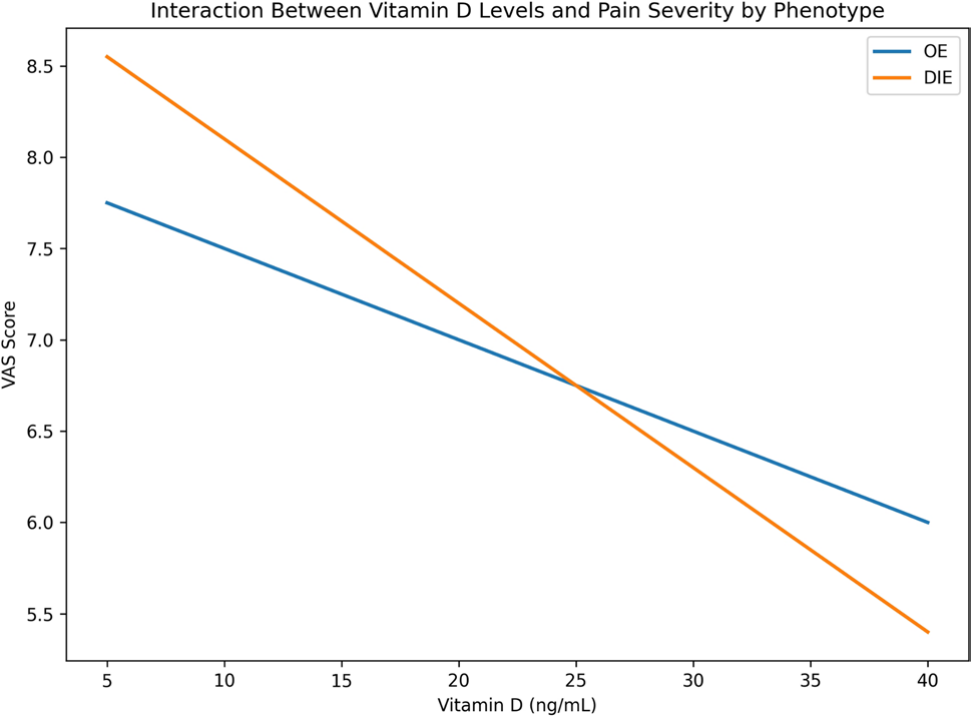
Interaction between vitamin D levels and pain severity by phenotype. Graph demonstrating the interaction between serum 25-hydroxyvitamin D [25(OH)D] levels and endometriosis phenotype on pain severity. The inverse association between vitamin D levels and VAS scores is more pronounced in the DIE group compared to the OE group. This interaction suggests that the association between lower vitamin D levels and higher pain intensity differs by phenotype and is more evident in patients with deep infiltrating endometriosis (interaction *p* = 0.004)

After adjustment, disease stage was not significantly associated with pain outcomes (*p* = 0.12), whereas the association between 25(OH)D levels and pain remained statistically significant. A significant interaction between vitamin D levels and phenotype was observed (interaction *p* = 0.004), indicating that the association between lower vitamin D levels and higher pain scores differed by phenotype and appeared more pronounced in patients with the DIE phenotype (Fig. 4).

### ROC analysis

**Fig. 5:**
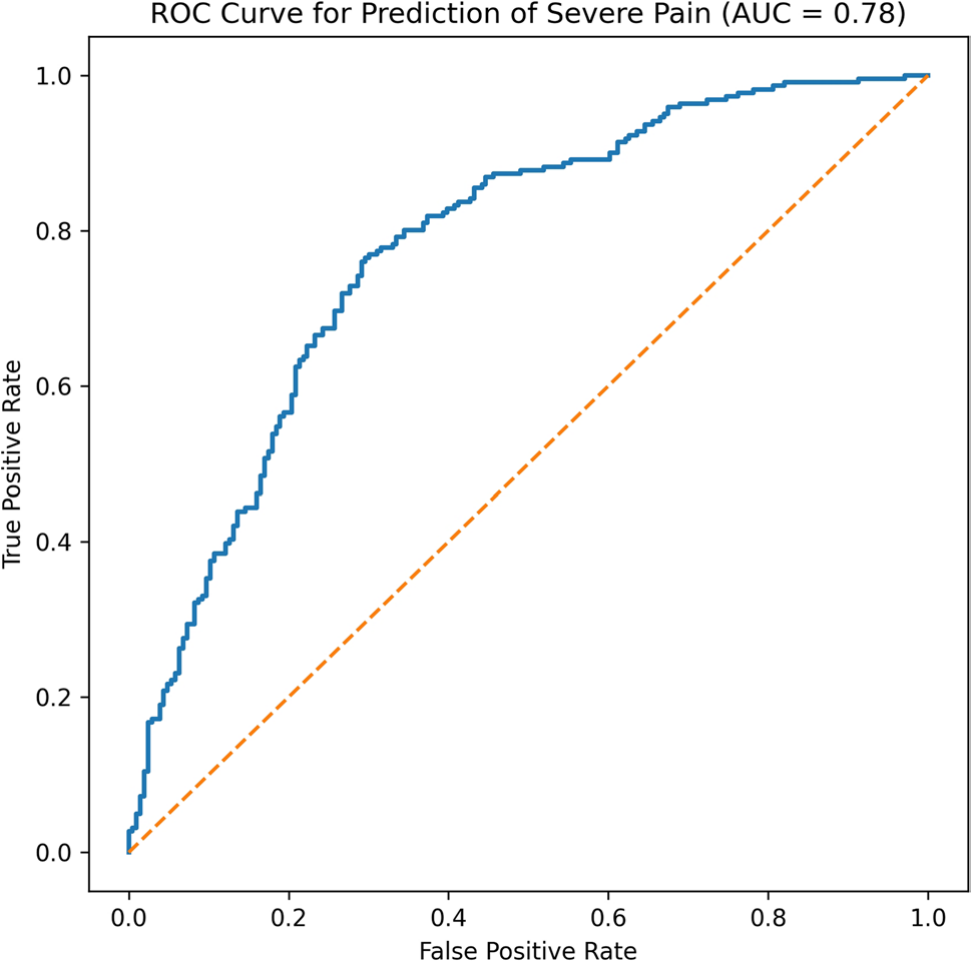
ROC curve analysis for identification of severe pelvic pain. Receiver operating characteristic (ROC) curve evaluating the performance of serum 25-hydroxyvitamin D [25(OH)D] levels in identifying severe pelvic pain (VAS ≥ 7). The area under the curve (AUC) was 0.74 (95% CI: 0.69–0.79, *p* < 0.001), indicating moderate discriminatory ability. The optimal cutoff value demonstrated acceptable sensitivity and specificity

Receiver operating characteristic (ROC) analysis showed that serum 25(OH)D levels were associated with severe pelvic pain (VAS ≥ 7), with an area under the curve (AUC) of 0.74 (95% CI: 0.69–0.79, *p* < 0.001) (Fig. 5; Table 5).

**Table 5:** Diagnostic Performance of Serum Vitamin D Levels for Identifying Severe Pelvic Pain (VAS ≥ 7)

Parameter	Value
AUC (ROC)	0.74 (95% CI: 0.69–0.79)
Cutoff (ng/mL)	≤ 18.5
Sensitivity (%)	72.3
Specificity (%)	68.1
PPV (%)	70.5
NPV (%)	69.8
*p*-value	< 0.001

Receiver operating characteristic (ROC) analysis was performed to evaluate the ability of serum vitamin D levels to identify severe pelvic pain (VAS ≥ 7). The optimal cutoff value was determined using the Youden index

*ROC* receiver operating characteristic, *AUC *area under the curve, * PPV *positive predictive value, *NPV*negative predictive value, * VAS* Visual Analog Scale

The optimal cut-off value was identified as ≤ 18.5 ng/mL, corresponding to a sensitivity of 72.3% and a specificity of 68.1%. Subgroup analyses suggested that the discriminatory performance of this threshold was more pronounced in patients with the DIE phenotype. Seasonal variation was not significantly associated with pain outcomes and was not retained in the final multivariable model.

## Discussion

The present study shows that lower serum 25-hydroxyvitamin D [25(OH)D] levels are significantly associated with greater pain severity in women with endometriosis. Notably, this association was not uniform across all patients but appeared more pronounced in those with the deep infiltrating endometriosis (DIE) phenotype. This phenotype-specific interaction suggests that the relationship between vitamin D status and pain may vary according to underlying disease characteristics.

The relationship between vitamin D and endometriosis has been widely investigated; however, findings remain inconsistent. While several studies have reported lower vitamin D levels in patients with endometriosis and possible associations with disease presence [10–12], others have not demonstrated a significant relationship [11, 12]. Genetic polymorphisms related to vitamin D receptor function have also been investigated in endometriosis, although findings remain inconsistent [13]. One potential explanation for these discrepancies is the limited consideration of disease heterogeneity in prior studies. The phenotype-based approach used in the present study may contribute to a more nuanced interpretation of these inconsistencies, suggesting that vitamin D levels may be differentially associated with clinical manifestations, such as pain, rather than disease presence alone.

In this study, lower vitamin D levels were consistently associated with higher scores for dysmenorrhea, chronic pelvic pain, and dyspareunia. These findings are in line with previous reports suggesting that vitamin D may be associated with pain perception, potentially through its role in inflammatory pathways [14, 15]. Vitamin D has been reported to influence the expression of pro-inflammatory cytokines such as IL-1β, IL-6, and TNF-α, which are implicated in pain sensitization; however, the extent to which these mechanisms are directly involved in endometriosis-related pain remains uncertain.

The more pronounced association observed in the DIE phenotype may reflect its distinct pathophysiological features. DIE lesions are characterized by deep tissue invasion, increased inflammatory activity, higher nerve fiber density, and enhanced neuroangiogenic signaling, all of which are associated with more severe pain symptoms [15–21]. In this context, lower vitamin D levels may be linked to a more pronounced inflammatory or neurobiological profile; however, this interpretation should be considered hypothesis-generating rather than confirmatory.

Previous studies evaluating the effect of vitamin D on pain have yielded conflicting results. While some clinical studies and systematic reviews have reported reductions in pelvic pain with vitamin D or antioxidant supplementation, findings are not consistent across studies or pain domains [4, 22–27]. Variations in study design, patient populations, and the absence of phenotype-based analyses may contribute to these differences. The consistent associations observed across pain domains in the present study may partly relate to the phenotype-based classification; however, this does not establish a causal relationship.

The observed interaction between vitamin D levels and phenotype underscores the importance of accounting for disease heterogeneity in endometriosis research. Rather than indicating a uniform effect, the findings suggest that the association between vitamin D status and pain may be context-dependent and potentially more evident in certain phenotypic subgroups such as DIE. These findings are also supported by previous studies highlighting variability in clinical presentation and biomarker profiles across endometriosis phenotypes [28–31].

From a clinical perspective, these findings suggest that serum vitamin D levels may be associated with the burden of pain symptoms, particularly in patients with DIE. However, these results should be interpreted with caution, and they do not support the use of vitamin D levels as a standalone biomarker or as a basis for therapeutic decision-making in pain management. Previous observational and registry-based studies have also emphasized the need for individualized approaches in endometriosis management [32–34]. Therefore, vitamin D should not be interpreted as an independent causal factor, but rather as a potential marker within a multifactorial and complex clinical context.

The ROC analysis demonstrated a moderate discriminatory ability of vitamin D levels for identifying severe pain. This suggests that vitamin D alone may have limited utility as an independent marker but could potentially contribute to broader risk stratification models when combined with other clinical variables. Similar moderate diagnostic performances have been reported in imaging and biomarker-based approaches [35, 36].

Several limitations should be considered. First, the retrospective and cross-sectional nature of the study precludes any inference of causality or temporal relationships. Second, important lifestyle-related factors influencing vitamin D levels—such as sun exposure, dietary habits, physical activity, and socioeconomic status—were not available in the dataset. Although seasonal variation was considered, it does not fully account for interindividual variability, and residual confounding cannot be excluded. Third, pain assessment was based on the VAS, which is inherently subjective, although the evaluation of multiple pain domains partially mitigates this limitation. The ongoing search for reliable biomarkers in endometriosis has been highlighted in recent reviews [37].

Despite these limitations, the study has notable strengths. The relatively large sample size and the inclusion of surgically and histopathologically confirmed cases enhance internal validity. In addition, the phenotype-based analytical approach, including interaction modeling, provides a more detailed evaluation of the association between vitamin D levels and pain compared to previous studies.

In conclusion, lower serum vitamin D levels are associated with increased pain severity in women with endometriosis, with a stronger association observed in the DIE phenotype. These findings support the need for further prospective and interventional studies to clarify the nature of this association and its potential clinical relevance.

## Conclusion

This study demonstrates a significant inverse association between serum 25-hydroxyvitamin D levels and pain severity in women with endometriosis. Lower vitamin D levels were consistently associated with higher scores for dysmenorrhea, chronic pelvic pain, and dyspareunia.

This association was not uniform across all patients and appeared more pronounced in those with the deep infiltrating endometriosis (DIE) phenotype, underscoring the heterogeneous nature of the disease.

Although vitamin D levels showed moderate discriminatory ability for identifying severe pain, they are unlikely to serve as a standalone marker and may instead provide complementary information within a broader clinical context.

Given the retrospective and cross-sectional nature of the study, causal relationships cannot be established. Further prospective and interventional studies are needed to better understand the nature of this association and its potential clinical implications in the management of endometriosis-related pain.

## Data Availability

The datasets used and/or analyzed during the current study are available from the corresponding author on reasonable request.

## Abbreviations

25(OH)D: 25-hydroxyvitamin D
AUC: Area under the curve
BMI: Body mass index
CA125: Cancer antigen 125
CI: Confidence interval
CPP: Chronic pelvic pain
DIE: Deep infiltrating endometriosis
GnRH: Gonadotropin-releasing hormone
IQR: Interquartile range
NRS: Numeric Rating Scale
OE: Ovarian endometrioma
PPV: Positive predictive value
NPV: Negative predictive value
rASRM: Revised American Society for Reproductive Medicine
ROC: Receiver operating characteristic
SPSS: Statistical Package for the Social Sciences
VAS: Visual Analog Scale
VDBP: Vitamin D binding protein
VDR: Vitamin D receptor

## References

[1] Zondervan KT, Becker CM, Missmer SA, Endometriosis. N Engl J Med. 2020;382(13):1244–56. 10.1056/NEJMra1810764. 32212520 10.1056/NEJMra1810764

[2] Malutan AM, Drugan T, Costin N, et al. Pro-inflammatory cytokines for evaluation of inflammatory status in endometriosis. Cent Eur J Immunol. 2015;40(1):96–102. 10.5114/ceji.2015.50840. 26155190 10.5114/ceji.2015.50840 PMC4472546

[3] Zhang M, Li X, Zhang Y, et al. Endometriosis: Pathogenesis, signaling pathways, and therapeutic targets. Int J Mol Sci. 2023;24(11):9472. 10.3390/ijms24119472. 37298423 10.3390/ijms24119472 PMC10253590

[4] Layanun V, Somboonporn W, Aupongkaroon P, et al. Analysis of Vitamin D and VDR Expression in Women with Advanced Endometriosis: A Case-Control Study in Thailand. Biomedicines. 2025;13(7):1605. 10.3390/biomedicines13071605. 40722678 10.3390/biomedicines13071605 PMC12292792

[5] Baradwan S, Gari A, Sabban H, et al. The effect of antioxidant supplementation on dysmenorrhea and endometriosis-associated painful symptoms: a systematic review and meta-analysis of randomized clinical trials. Obstet Gynecol Sci. 2024;67(2):186–98. 10.5468/ogs.23210. 38221738 10.5468/ogs.23210 PMC10948216

[6] Anastasi E, Fuggetta E, De Vito C, et al. Low levels of 25-OH vitamin D in women with endometriosis and associated pelvic pain. Clin Chem Lab Med. 2017;55(12):e282–4. 10.1515/cclm-2017-0382. 28453438 10.1515/cclm-2017-0016

[7] Coxon L, Demetriou L, Vincent K. Current developments in endometriosis-associated pain. Cell Rep Med. 2024;5(10):101769. 10.1016/j.xcrm.2024.101769. 39413731 10.1016/j.xcrm.2024.101769 PMC11513828

[8] Chapron C, Marcellin L, Borghese B, Santulli P. Rethinking mechanisms, diagnosis and management of endometriosis. Nat Rev Endocrinol. 2019;15(11):666–82. 10.1038/s41574-019-0245-z. 31488888 10.1038/s41574-019-0245-z

[9] Hawker GA, Mian S, Kendzerska T, French M. Measures of adult pain: Visual Analog Scale for Pain (VAS Pain), Numeric Rating Scale for Pain (NRS Pain), McGill Pain Questionnaire (MPQ), Short-Form McGill Pain Questionnaire (SF-MPQ), Chronic Pain Grade Scale (CPGS), Short Form-36 Bodily Pain Scale (SF-36 BPS), and Measure of Intermittent and Constant Osteoarthritis Pain (ICOAP). Arthritis Care Res (Hoboken). 2011;63(Suppl 11):S240–52. 10.1002/acr.20543. 22588748 10.1002/acr.20543

[10] Amro B, Ramirez Aristondo ME, Alsuwaidi S, et al. New Understanding of Diagnosis, Treatment and Prevention of Endometriosis. Int J Environ Res Public Health. 2022;19(11):6725. 10.3390/ijerph19116725. 35682310 10.3390/ijerph19116725 PMC9180566

[11] Xie B, Liao M, Huang Y, et al. Association between vitamin D and endometriosis among American women: National Health and Nutrition Examination Survey. PLoS ONE. 2024;19(1):e0296190. 10.1371/journal.pone.0296190. 38215179 10.1371/journal.pone.0296190 PMC10786361

[12] Jafari M, Khodaverdi S, Sadri M, et al. Association Between Vitamin D Receptor (VDR) and Vitamin D Binding Protein (VDBP) Genes Polymorphisms to Endometriosis Susceptibility in Iranian Women. Reprod Sci. 2021;28(12):3491–7. 10.1007/s43032-021-00598-z. 33948927 10.1007/s43032-021-00598-z

[13] Lee HH, Mun MJ, Kim TH, et al. Relationships between vitamin D receptor genetic polymorphisms and endometriosis in Korean women. Clin Exp Obstet Gynecol. 2019;46(6):876–80. 10.12891/ceog4686.2019.

[14] Jennings BS, Hewison M. Vitamin D and Endometriosis: Is There a Mechanistic Link? Cell Biochem Funct. 2025;43(1):e70037. 10.1002/cbf.70037. 39739404 10.1002/cbf.70037 PMC11686426

[15] Koninckx PR, Ussia A, Adamyan L, et al. Deep endometriosis: definition, diagnosis, and treatment. Fertil Steril. 2012;98(3):564–71. 10.1016/j.fertnstert.2012.07.1061. 22938769 10.1016/j.fertnstert.2012.07.1061

[16] Manieri Rocha R, Leonardi M, Eathorne A, et al. Anatomical distribution of endometriosis: A cross-sectional analysis of transvaginal ultrasound in symptomatic patients. Australas J Ultrasound Med. 2023;26(3):131–41. 10.1002/ajum.12327. 37701766 10.1002/ajum.12327 PMC10493340

[17] Kanti FS, Allard V, Métivier AA, et al. Pain Phenotypes in Endometriosis: A Population-Based Study Using Latent Class Analysis. BJOG. 2025;132(4):492–503. 10.1111/1471-0528.18021. 39627905 10.1111/1471-0528.18021 PMC11794060

[18] Abrao MS, Andres MP, Miller CE, et al. AAGL 2021 Endometriosis Classification: An Anatomy-based Surgical Complexity Score. J Minim Invasive Gynecol. 2021;28(11):1941–e19501. 10.1016/j.jmig.2021.09.709. 34583009 10.1016/j.jmig.2021.09.709

[19] Bourg J, Ruaux E, Bolze PA, et al. Pelvic nerve endometriosis: MRI features and key findings for surgical decision. Insights Imaging. 2025;16(1):131. 10.1186/s13244-025-02005-6. 40537672 10.1186/s13244-025-02005-6 PMC12179019

[20] Szubert M, Rogut M, Ziętara M, et al. Expression of nerve growth factor (NGF) in endometrium as a potential biomarker for endometriosis - Single tertiary care centre study. J Gynecol Obstet Hum Reprod. 2021;50(3):101895. 10.1016/j.jogoh.2020.101895. 32827836 10.1016/j.jogoh.2020.101895

[21] Taylor RN, Kane MA, Sidell N. Pathogenesis of Endometriosis: Roles of Retinoids and Inflammatory Pathways. Semin Reprod Med. 2015;33(4):246–56. 10.1055/s-0035-1554920. 26132929 10.1055/s-0035-1554920 PMC4745121

[22] Farhangnia P, Noormohammadi M, Delbandi AA. Vitamin D and reproductive disorders: a comprehensive review with a focus on endometriosis. Reprod Health. 2024;21(1):61. 10.1186/s12978-024-01797-y. 38698459 10.1186/s12978-024-01797-y PMC11064344

[23] Smolarz B, Szyłło K, Romanowicz H, Endometriosis. Epidemiology, Classification, Pathogenesis, Treatment and Genetics (Review of Literature). Int J Mol Sci. 2021;22(19):10554. 10.3390/ijms221910554. 34638893 10.3390/ijms221910554 PMC8508982

[24] Moini A, Yazdian Anaraki M, Shirzad N, et al. The effect of vitamin D supplementation on endometriosis-related pain: a randomized clinical trial. Int J Fertil Steril. 2016;10(3):316–23. 10.22074/ijfs.2016.5110.

[25] Şanlıkan F, Bağlar İ, Keleş E, Mat E, Öztürk UK, Birge Ö. Can hematological and biochemical parameters clinically predict the diagnosis of deep infiltrating endometriosis? BMC Women’s Health. 2026;26:8. 10.1186/s12905-025-04197-x. 10.1186/s12905-025-04197-x PMC12771800 41318478

[26] Zhong Q, Jin Z, Ma J, et al. 1,25-Dihydroxy vitamin D3 inhibits LPS-mediated inflammatory responses in endometriosis. Ann Med. 2025;57(1):2523563. 10.1080/07853890.2025.2523563. 40579858 10.1080/07853890.2025.2523563 PMC12207774

[27] Tsokkou S, Matsas A, Konstantinidis I, et al. Therapeutic Effects of Vitamins in Endometriosis Patients: A Systematic Review of Randomized Controlled Trials. Int J Mol Sci. 2026;27(3):1476. 10.3390/ijms27031476. 41683896 10.3390/ijms27031476 PMC12898241

[28] Liu D, Liu M, Yu P, Li H. Brain-derived neurotrophic factor and nerve growth factor expression in endometriosis: A systematic review and meta-analysis. Taiwan J Obstet Gynecol. 2023;62(5):634–9. 10.1016/j.tjog.2023.07.003. 37678988 10.1016/j.tjog.2023.07.003

[29] Sommar A, Bahat PY, Özaydin IY, et al. Influence of endometrial nerve fibers and hormones on pain in women with endometriosis. Eur J Obstet Gynecol Reprod Biol. 2025;310:113950. 10.1016/j.ejogrb.2025.113950. 40184895 10.1016/j.ejogrb.2025.113950

[30] Zhang R, Lv H, Liu J, et al. The impact of MTHFR and VDR polymorphisms on endometriosis susceptibility: Insights from a systematic review and meta-analysis. J Reprod Immunol. 2025;168:104449. 10.1016/j.jri.2025.104449. 39946760 10.1016/j.jri.2025.104449

[31] Riccio LG, Santulli P, Marcellin L, et al. Immunology of endometriosis. Best Pract Res Clin Obstet Gynaecol. 2018;50:39–49. 10.1016/j.bpobgyn.2018.01.010. 29506962 10.1016/j.bpobgyn.2018.01.010

[32] Ng CHM, Michelmore AG, Mishra GD, et al. Establishing the Australian National Endometriosis Clinical and Scientific Trials (NECST) Registry: A protocol paper. Reprod Fertil. 2023;4(2):e230014. 10.1530/RAF-23-0014. 37224076 10.1530/RAF-23-0014 PMC10305626

[33] Parasar P, Ozcan P, Terry KL. Endometriosis: epidemiology, diagnosis and clinical management. Curr Obstet Gynecol Rep. 2017;6(1):34–41. 10.1007/s13669-017-0187-1. 29276652 10.1007/s13669-017-0187-1 PMC5737931

[34] Zondervan KT, Becker CM, Koga K, et al. Endometr Nat Rev Dis Primers. 2018;4(1):9. 10.1038/s41572-018-0008-5. 10.1038/s41572-018-0008-5 30026507

[35] Keckstein J, Hoopmann M, Merz E, et al. Expert opinion on the use of transvaginal sonography for presurgical staging and classification of endometriosis. Arch Gynecol Obstet. 2023;307(1):5–19. 10.1007/s00404-022-06766-z. 36367580 10.1007/s00404-022-06766-z PMC9837004

[36] Hofbeck L, Au K, Blum S, et al. Clinical characterization of endometriosis phenotypes. Arch Gynecol Obstet. 2025;312(6):2089–100. 10.1007/s00404-025-08191-4. 41107502 10.1007/s00404-025-08191-4 PMC12705834

[37] Encalada Soto D, Rassier S, Green IC, et al. Endometriosis biomarkers of the disease: an update. Curr Opin Obstet Gynecol. 2022;34(4):210–9. 10.1097/GCO.0000000000000798. 35895963 10.1097/GCO.0000000000000798

